# Transformations and Transmission – A Contextual Look at Religious Biographies of Older Adults

**DOI:** 10.1093/geroni/igab046.3341

**Published:** 2021-12-17

**Authors:** Jenni Spannari, Kati Tervo-Niemela, Laura Kallatsa

**Affiliations:** University of Eastern Finland, Joensuu, Pohjois-Karjala, Finland

## Abstract

Examination of religious biographies tend to show increase of religiosity towards old age, most often in the context of a previously familiar religious community. These changes in individuals do not happen in a vacuum. Religious landscapes are also in transformation, characterized by a steady decline of institutional religiosity and religious practice in most European countries and more recently in the US and Canada, too. However, there is a dire lack of detailed knowledge on how these changes in individuals and societies are intertwined. This paper presents findings of the Finnish sub-project of the five-country research project “Transmission of religion across generations.” The paper utilizes both three-generation interviews, and the contextual information gathered in the families about the past and present generations. The narratives about religious biographies of the oldest (gen.1) interviewees are discussed, and set in the context of the changes in the surrounding social sphere and the interviewees’ role in the family. Key findings include a general trend of increased flexibility, openness and communication over time – both in the religious views of the individuals, the roles different generations take in the family, and the cultural atmosphere in the society. Also, the results suggest that this flexibility is an essential factor in successful transmission of religion or other convictions across generations. The findings illustrate the complexity and contextuality of building and researching narratives of religious biographies. Thus, the results contribute to future examinations on how changes in societies and families affect the religious styles and convictions of older adults.

